# Supplementary material for: Isolation and Characterization of Cell-Free DNA from Cerebral Organoids
Source: Int J Mol Sci. 2024 May 18;25(10):5522. doi: 10.3390/ijms25105522 (PMC11121789; doi:10.3390/ijms25105522)

Supplementary Figure S1: Sources of cfDNA in Cerebral Organoids During Growth.
Proportion of cfDNA sequencing reads contributed by rodent and human sources on growth days 10, 21, and 41.

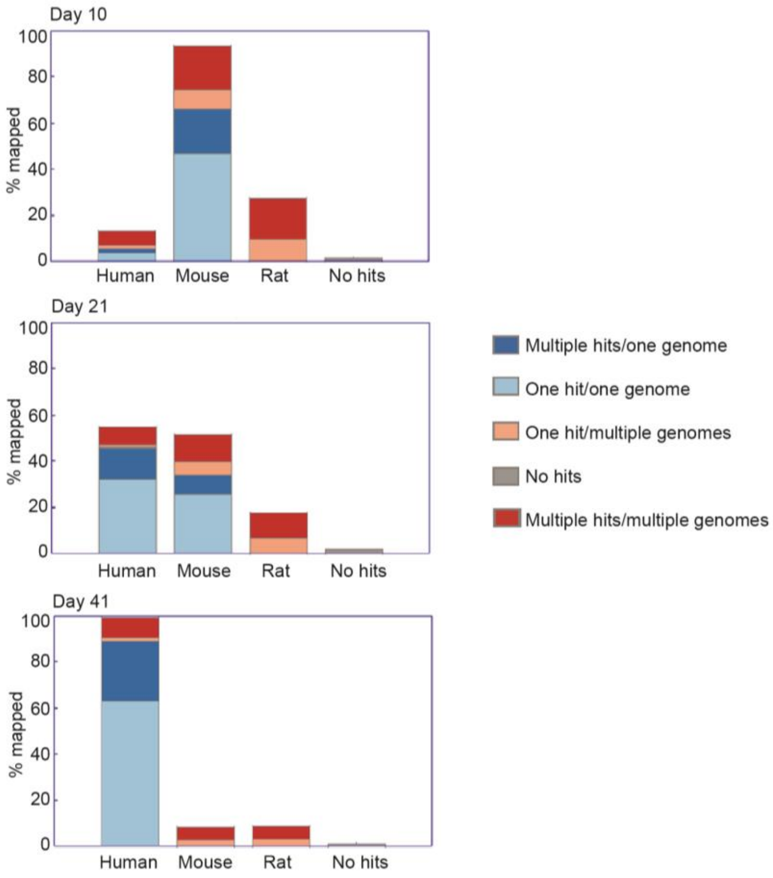

Supplementary Figure S2: Definition of regions of interest (ROI) in cerebral organoid-derived cfDNA sequencing data. Shown is a representative view of sequenced reads in cfDNA samples derived from cerebral organoids (CO), Macs2-identified peaks, intersection of peaks present in all three replicates, and union of peaks (ROI)

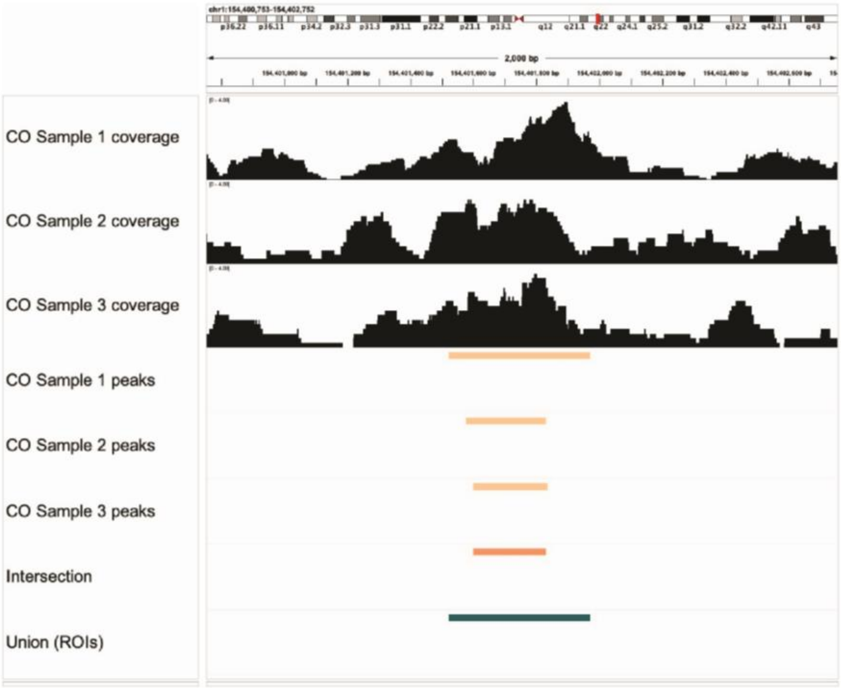

Supplement: Supplementary file 1 [file ijms-25-05522-s001.zip › ijms-3003147-supplementary.pdf]
